# Tetrahydrodipicolinate N-Succinyltransferase and Dihydrodipicolinate Synthase from *Pseudomonas aeruginosa*: Structure Analysis and Gene Deletion

**DOI:** 10.1371/journal.pone.0031133

**Published:** 2012-02-16

**Authors:** Robert Schnell, Wulf Oehlmann, Tatyana Sandalova, Yvonne Braun, Carmen Huck, Marko Maringer, Mahavir Singh, Gunter Schneider

**Affiliations:** 1 Department of Medical Biochemistry and Biophysics, Karolinska Institutet, Stockholm, Sweden; 2 LIONEX Diagnostics and Therapeutics, Braunschweig, Germany; 3 mfd Diagnostics GmbH, Wendelsheim, Germany; University of Canterbury, New Zealand

## Abstract

The diaminopimelic acid pathway of lysine biosynthesis has been suggested to provide attractive targets for the development of novel antibacterial drugs. Here we report the characterization of two enzymes from this pathway in the human pathogen *Pseudomonas aeruginosa*, utilizing structural biology, biochemistry and genetics. We show that tetrahydrodipicolinate N-succinyltransferase (DapD) from *P. aeruginosa* is specific for the L-stereoisomer of the amino substrate L-2-aminopimelate, and its D-enantiomer acts as a weak inhibitor. The crystal structures of this enzyme with L-2-aminopimelate and D-2-aminopimelate, respectively, reveal that both compounds bind at the same site of the enzyme. Comparison of the binding interactions of these ligands in the enzyme active site suggests misalignment of the amino group of D-2-aminopimelate for nucleophilic attack on the succinate moiety of the co-substrate succinyl-CoA as the structural basis of specificity and inhibition. *P. aeruginosa* mutants where the *dapA* gene had been deleted were viable and able to grow in a mouse lung infection model, suggesting that DapA is not an optimal target for drug development against this organism. Structure-based sequence alignments, based on the DapA crystal structure determined to 1.6 Å resolution revealed the presence of two homologues, PA0223 and PA4188, in *P. aeruginosa* that could substitute for DapA in the *P. aeruginosa* PAO1Δ*dapA* mutant. *In vitro* experiments using recombinant PA0223 protein could however not detect any DapA activity.

## Introduction

*Pseudomonas aeruginosa* is an opportunistic pathogen that causes persistent infections in humans. The limited reservoir of antibacterial drugs against gram-negative bacteria, inherent difficulties in the clinical treatment of *P. aeruginosa* infections and the onset of antibiotic resistance [1] emphasize the need to identify novel drug targets against these pathogens. Randomized transposon mutagenesis screens [2] had indicated several genes from the diaminopimelate (DAP) biosynthetic pathway to lysine in *P. aeruginosa* as essential, suggesting the corresponding gene products as potential targets for new antibacterial drugs. Similar findings were reported for other bacterial pathogens, such as *Mycobacterium tuberculosis* [3]–[5] and *Salmonella typhymurium* [6]. These observations reinforced previous notions that this pathway is a particular attractive target for the design of antibacterial compounds, as it is not found in humans and provides two essential metabolites, the amino acid lysine and its precursor, *meso*-diaminopimelate, a component of the cell wall peptidoglycan in bacteria [3], [7].

**Figure 1:**
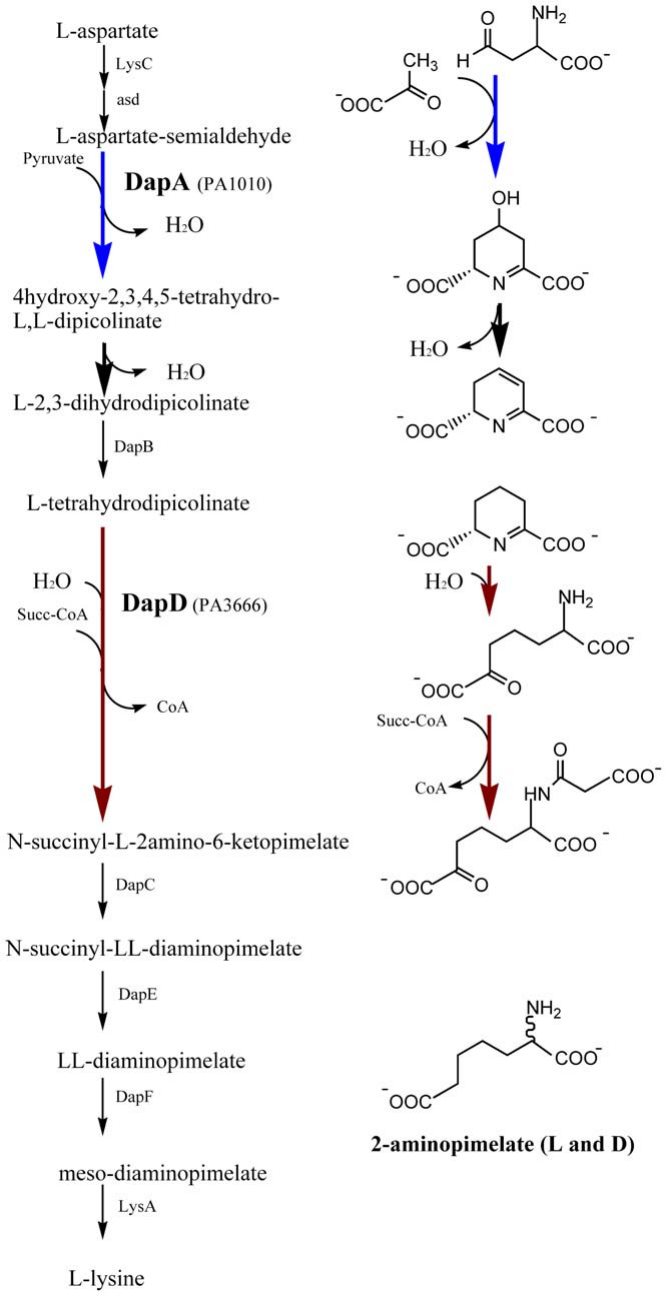
The diaminopimelic acid pathway of lysine biosynthesis. Left: the steps of the pathway are shown with the reactions catalyzed by DapA and DapD indicated by blue and red arrows, respectively. Right: Reactions catalyzed by dihydrodipicolinate synthase (DapA, PA1010) and tetrahydrodipicolinate N-succinyltransferase (DapD, PA3666). The structure of the substrate analogue 2-aminopimelate used as DapD substrate in this study is shown for comparison.

Dihydrodipicolinate synthase (DapA) catalyzes the first committed step of the DAP pathway, the condensation of L-aspartate-semialdehyde and pyruvate to 4-hydroxy-2,3,4,5-tetrahydro-L,L-dipicolinic acid, which is non-enzymatically dehydrated to yield L-2,3-diydrodipicolinate [7], [8] (Figure 1). The reaction mechanism involves a covalent intermediate formed between the substrate pyruvate and an invariant lysine residue at the active site [8]. The crystal structure of *Escherichia coli* DapA revealed a homotetrameric enzyme, with the subunits displaying the ubiquitous β/α barrel fold [9]. By now structures of DapA from over 20 organisms have been determined and the majority all show a homotetrameric arrangement of the subunits [9]–[17]. However, functional homodimeric forms of DapA have also recently been described [18]–[20]. DapA is subject to allosteric feed-back regulation by L-lysine and crystal structures of the enzyme from *E. coli* and *P. aeruginosa* with bound lysine have revealed the structural basis for its regulation [12], [20]. The role of the active residues Lys-161, Thr-44, Arg-138 in catalysis has been investigated for *Escherichia coli* DapA identifying the proton-relay system and the mode of enzyme-substrate interactions [21]–[23].

Tetrahydrodipicolinate N-succinyltransferase (DapD) catalyses the transfer of the succinyl moiety of succinyl-CoA to the α-amino group of tetrahydrodipicolinate, the first committed step in the succinylase branch of the DAP biosynthesis pathway present in most Gram-negative bacteria and mycobacteria [24] (Figure 1). The crystal structures of DapD from *Escherichia coli* [25], *Mycobacterium tuberculosis* [26] and *Mycobacterium bovis* [27] have been published, although the origin of the gene for the latter has been questioned [26]. In addition, the coordinates for DapD from *Campylobacter jejuni* (2RIJ), *Enterococcus feacalis* (3CJ8), *Brucella melitensis* (3EG4), and *Yersinia pestis* (3GOS) have been deposited in the Protein Data Bank by several Structural Genomics projects. In all these cases, DapD reveals a homotrimeric structure with the three active sites located between adjacent subunits. The common characteristic structural feature of DapD enzymes is a left-handed parallel β-helix (LβH) domain. This trimeric structural module occurs in many acyltransferases and provides binding surfaces for the substrate acyl-CoA. DapDs typically contain additional N- and C-terminal domains fused to the central LβH domain.

Crystallographic investigations of the binary complex of *Mt*DapD with succinyl-CoA [26] and of ternary complexes of DapD [28], [29] are consistent with a nucleophilic attack of the substrate amino group on the carbonyl group of succinyl-CoA. Comparisons of the structures of apo-DapD with that of ligand bound DapD revealed conformational changes upon binding of the substrates that involve several regions of the polypeptide chain [25], [28], [29].

As *dapA* (PA1010) and *dapD* (PA3666) were suggested to be essential genes in *P. aeruginosa* [2] we set out to characterize these enzymes of the DAP pathway in this organism in more detail. Furthermore we characterized a gene knockout both *in vitro* and *in vivo* using a mouse model of acute *P. aeruginosa* infection. We show that *Pa*DapD is specific for L-aminopimelic acid (L-2AP), and that the stereoisomer D-2-aminopimelic (D-2AP) acid is not a substrate, but a weak inhibitor. The crystal structures of *Pa*DapD with L-2AP and D-2AP, respectively, reveal the structural basis for inhibition and stereoselectivity. The *in vivo* knockout studies show that *P. aeruginosa* mutants lacking the *dapA* gene are still able to cause acute infection in mice, suggesting that this enzyme may not be a suitable target for new antibiotics against this organism.

## Materials and Methods

### Construction of suicide vectors for knock out of *dapA* (PA1010)

For gene replacement of *dapA* (PA1010) the s*acB*-based strategy [30], [31] was employed. In order to construct the suicide vectors for deletion of *dap*A (PA1010) regions of approximately 400 bp flanking the target genes were amplified using genomic DNA prepared from *Pseudomonas aeruginosa* PAO1 (ATCC 47085) as template. Primers 404PA1010 (5′-GCGACGGAATTCGGGCCGGCAGGCTGCCCT-3′) with an *Eco*RI site at the 5′-end and 405PA1010 (5′-GCGACGGGATCCGCAACCGCTCCTGCCGCG-3′) containing a *Bam*HI site were used for amplification of the upstream fragment of the *dap*A coding sequence. Primers 406PA1010 (5′-GCGACGGGATCCTCAAGGAGCTCCACGCAA-3′) containing a *Bam*HI site and 407PA1010 (5′-GCGACGAAGCTTCGGAAACCGTTCTCCTCG-3′) with a *Hind*III site were used for downstream fragment amplification. After digest with the corresponding endonucleases both fragments were inserted into the multiple cloning site of vector pEX18Ap [32]. The Gentamicin resistance cassette of pPS858 [32] was excised using *Bam*HI and introduced into the *Bam*HI and *Bgl*II site, respectively, between the two flanking fragments. Constructs were confirmed by sequencing and then transformed into the *E. coli* donor strain ST18 that was used for conjugational transfer of the plasmids into *P. aeruginosa* PAO1 as described previously [31].

After counter-selection on LB-agar plates containing 5% sucrose and 80 µg/mL gentamycin, the obtained clones were tested for carbenicillin sensitivity by replica plating. In order to confirm the loss of plasmid borne DNA due to recombination events, total DNA was isolated from the potential KO mutants and used in PCR together with primers specific for internal sequences of *sacB* and *bla* genes. Additionally the correct replacement of the target gene by the gentamycin cassette was confirmed also by PCR and sequencing. Primers located in approximately 200 base pairs distance from the ends of the cassette and directed outwards were used in combination with primers located about 500 base pairs up- and downstream, respectively, from the target gene. The resulting PCR products were isolated and sequenced.

### Agarose bead preparation for infection with *P. auriginosa* PAO1 wt and *P. aeruginosa* PAO1Δ*dapA*

Agarose beads were prepared using a method described previously [33] with some modifications. *P. aeruginosa* PAO1 (ATCC 47085) and *P. aeruginosa* PAO1Δ*dapA* strains were cultured over night at 37°C in LB and LB+Gentamycin (80 µg/ml), respectively. After centrifugation at 4.000 rpm for 10 min the sedimented bacteria were resuspended in 1 ml sterile PBS (pH 7.4) and added to 5 ml 2% Agarose prewarmed to 50°C. The bacteria-agarose mixture was transferred rapidly to 5 ml 50°C prewarmed heavy white mineral oil. After intensive vortexing the mixture was cooled on ice for 5 minutes followed by centrifugation at 4.000 rpm for 10 min. The resulting agarose beads were washed three times in sterile PBS (pH 7.4). The load of bacteria in the agarose beads was quantified by plating 10-fold serial dilutions on Columbia blood agar plates. The inoculums for infection were prepared by diluting the bead suspension with PBS (pH 7.4) to 2×10^8^ CFU/ml.

### Lung infection of mice

For the infection studies we used female NMRI outbred mice at the age of min. 6 weeks. The animals were anesthetized using a Ketamin-Xylazin mixture applied by i.p. injection. Mice were intratracheally infected with 50 µl of the bead suspension at a concentration of 2×10^8^ CFU/ml resulting in 1×10^7^ CFU. This study was carried out in strict accordance with the recommendations of the European Commission and the law for the Care and Use of Laboratory Animals of the Government of the Federal Republic of Germany. The protocol was approved by the Committee on the Ethics of Animal Experiments of the Landesuntersuchungsamt of the government of Rheinland-Pfalz, Federal Republic of Germany (Permit Number: 23 177-07/G09-15-001).

### Quantification of bacteria in the lung

72 hours after infection, the mice were sacrificed by CO_2_ inhalation. Lungs were isolated and homogenized through microfilters. Mouse lung homogenates were diluted 1∶1, 1∶10 and 1∶100 with sterile PBS (pH 7.4). The different dilutions were plated on Columbia blood agar plates in case of *P. aeruginosa* PAO1 wildtype, and on agar plates containing Gentamycin for *P. aeruginosa* PAO1Δ*dapA*. After overnight incubation at 37°C colony counts were determined for each individual dilution.

### Gene cloning, protein expression and purification

The DNA sequence coding for DapD (PA3666) from *P. aeruginosa* PAO1 genomic DNA template (ATCC 47085D) was amplified by PCR and cloned in the expression vector pET28a (Novagen) using upstream *Nde*I and downstream *Hind*III sites resulting in a cleavable six-histidine tag at the N-terminus. In this construct, thrombin cleavage results in a recombinant protein with three additional amino acids (Gly-Ser-His) at the N-terminus. The genes coding for DapA (PA1010) and its homologues PA0223 and PA4188 were amplified by PCR and cloned in pNIC28Bsa4 (GenBank Accession No. EF198106) using the LIC method resulting in an N-terminal His6-tag with a TEV protease cleavage site. Similarly, the expression construct of DapB (dihydropicolinate reductase, DHDPR) from *E. coli* was based on pNIC28Bsa4 providing an N-terminal hexahistidine tag. All expression constructs were confirmed by DNA sequencing.

*Escherichia coli* BL21(DE3) (Novagen) carrying the constructs pET-His6PA3666 or pNIC-His6PA1010 was cultivated in 1.5 L of LB medium supplemented with kanamycine (30 µg/ml) at 21°C. At an OD600 of 0.6–0.7 *dapA* or *dapD* gene expression was induced by the addition of IPTG to 0.1 mM. After approximately 24 hours the cells were harvested and then re-suspended in a buffer consisting of 10 mM Tris-HCl pH 8.0, 300 mM NaCl, and 10 mM imidazole. Cells were disrupted by freeze/thaw cycles, lysozyme and DNaseI treatment followed by sonication. The clarified lysates were loaded on a Ni-NTA column (Qiagen) with a column volume of 1.5 ml. Pure recombinant proteins were eluted in fractions containing 50–200 mM imidazole. The eluted proteins were desalted on a Sephadex G-25 column (GE-Healthcare) and retained in a buffer of 25 mM Tris-HCl pH 8.0, 150 mM NaCl. In the case of DapD the N-terminal His6-tag was removed by thrombin cleavage. This procedure resulted in homogeneous DapD as verified by SDS PAGE. The pure His6-DapD and the thrombin processed DapD proteins were concentrated to 26 mg/ml by an Amicon centrifugation device with a 10 kDa MW cut-off. Monomodal size distribution was confirmed by analyzing the sample on a native polyacrylamide gel. In the case of DapA the proteolytic cleavage by TEV protease did not remove the N-terminal His6-tag efficiently, and His6-tagged protein was therefore purified and used in all experiments. Aliquots of the protein preparations were flash-frozen in liquid nitrogen and stored at −80°C until further use. Analytical size exclusion chromatography was carried out using a superdex-200 10/30 column (GE Healthcare, Uppsala, Sweden) with an equilibration buffer of 25 mM Tris-HCl pH 8.0, containing 150 mM NaCl. The column was calibrated with chymotrypsinogen-A (25 kDa), ovalbumin (43 kDa), albumin (67 kDa), catalase (232 kDa) and ferritin (440 kDa).

PA0223 and E. coli DapB were purified with affinity chromatography utilizing the vector supplied N-terminal His-tag. Elution fractions containing the pure proteins were passed through a PD10 column (GE Healthcare, Uppsala, Sweden) and eluted using a buffer consisting of 25 mM Tris-HCl pH 8.0, and 150 mM NaCl.

### Enzyme assays

Purified recombinant DapD was assayed spectrophotometrically at 412 nm for succinyl-transferase activity by monitoring the formation of free coenzyme-A derivatized by dithio-nitrobenzoate [24] using L-2-aminopimelate as amino acid substrate. A racemic mixture of 2-aminopimelate was purchased from Sigma, whereas the pure enantiomeric forms of L- and D-2 aminopimelate were obtained from Prof. Philip Cohen, University of Dundee. Typically, the reactions were carried out in a total volume of 0.1 ml at 22°C. The reaction mixture contained 100 mM TRIS-HCl pH 7.5, 2 mM MgCl_2_, succinyl-CoA at 0.1 mM, DTNB at 2.0 mM and DapD at 0.25 µM final concentrations. L-2-aminopimelate was used in the range 0.5–15 mM and D-2-aminopimelate at 2–4 mM. For the inhibition assays concentrations of D-2AP of up to 70 mM were used. The reactions were started by the addition of succinyl-CoA, and velocities were derived from the initial slopes of the time courses recorded at 412 nm. Each measurement was carried out in triplicates.

The activity assays for PA1010 (DapA) and PA0223 were based on the coupled assay using *E. coli* DHDPR as described by Kaur et al. [20]. The measurements were carried out at 22°C in 100 µl total volumes in 50 mM Tris-HCl at pH 8.0. The reaction mixtures contained 1.5 µg/ml DapA, 23 µg/ml DHDPR, 0.2 mM NADPH and 5 mM pyruvate. The reactions were started by the addition of S-aspartate-semialdehyde (Glycosyn, New Zeeland) to 0.2 mM concentration. The absorbance was monitored at 340 nm in a JASCO V-650 spectrophotometer to record NADPH consumption. In the case of PA0223, enzyme concentrations of 1.5 µg/ml and 15 µg/ml were used.

### Crystallization and data collection

Crystallization screening was carried out using the vapor diffusion method and a Phoenix crystallisation robot. DapA crystals were grown in hanging drops containing 2 µl of protein solution (12.5 mg/ml) and 2 µl of reservoir solution (18% of PEG6000, 0.2 M MgCl_2_, 0.1 M TRIS-HCl, pH 7.6). The crystals used for diffraction analysis were cyo-protected by a short soak in a buffer identical to the mother liquor containing 30% PEG6000. Diffraction quality crystals of DapD were obtained by equilibrating a mixture of 2 µl of protein solution (26 mg/ml) and 2 µl of reservoir solution (19–20% of PEG3350, 0.3–0.4 M succinate, pH 6.2) against the reservoir solution in hanging drops. Single rod-shaped crystals appeared after 24–48 hours. These were flash-frozen in liquid nitrogen without addition of any cryoprotectant. The crystals of the CoA complex were produced by co-crystallization in conditions identical to that of the apo-protein by adding CoA (final concentration 10–15 mM) to the protein solution for 10 minutes prior to crystallization. The crystals were isomophous with those obtained for the apo-enzyme. Incubation of *Pa*DapD with formyl-CoA for 15 minutes resulted in crystals that belong to the monoclinic space group (P2_1_). These crystals diffracted reproducibly to higher resolution. The complexes with L-2-aminopimelate and D-2-aminopimelate were obtained by soaking the monoclinic crystals in crystallization liquor containing 0.4 M of either D- or L-2-aminopimelate instead of succinate.

X-ray data to 2.95 Å for the DapD apo-enzyme were collected at the beamline ID14:4 of the European Synchrotron Radiation Facility (ESRF, Grenoble, France) at 110 K. The dataset for the binary complex DapD-CoA was collected at beamline ID14:1 (ESRF, Grenoble, France) at 110 K to 2.4 Å resolution. The X-ray data were processed and scaled with the programs MOSFLM and SCALA from the CCP4 suite [34]. These crystals belong to the tetragonal space group P4_1_2_1_2 with cell dimensions *a* = *b* = 122.4 Å, and *c* = 199.3 Å. A second dataset for the apo-enzyme was collected from the monoclinic crystal form at beamline I911-3 at MaxLab (Lund, Sweden) to 1.8 Å resolution. The diffraction datasets for the D- and the L-2-aminopimelate complexes were collected at MaxLab (Lund, Sweden) at the beamline I911-2 to 1.9 Å and at I911-3 to 2.5 Å resolution, respectively (table 1). The datasets scaled well in the orthorhombic Laue group P222, but a more detailed analysis indicated crystal twinning. The true space group is P2_1_, with a twinning operator (-h, -k, l). The twinning fractions vary between crystals from 0.35 to 0.43.

**Table 1:** Data collection and refinement statistics of the crystal structures of *Pa*DapD and *Pa*DapA.

Protein	DapA	DapD-CoA	DapD-Apo	DapD-D-AP	DapD-L-AP
PDB code/file	3QZE	3R5C	3R5D	3R5A	3R5B
Space group	P1	P4_1_2_1_2	P2_1_	P2_1_	P2_1_
**Cell**					
a,	43.1	123.3	83.0	83.3	82.7
b,	51.7	123.3	102.0	101.9	100.4
c (Å)	140.3	198.8	134.9	135.7	133.6
α, β, γ (°)	95.1, 90.0, 113.3	90.0, 90.0, 90.0	90.0, 90.0, 90.0	90.0, 90.0, 90.0	90.0, 90.1, 90.0
Resolution (Å)	40.0-1.59	66.2-2.40	25-1.80	22-1.88	37-2.50
High Resolution shell	1.67-1.59	2.53-2.40	1.90-1.80	1.98-1.88	2.64-2.50
No. of unique reflections	139082 (20245)	60616 (8713)	190821 (18894)	173938 (19600)	72498 (9568)
I/s(I)	9.5 (3.1)	17.1 (2.6)	10.8 (2.8)	9.8 (2.3)	6.7 (3.2)
Redundancy	2.0 (2.0)	6.6 (6.7)	2.6 (1.9)	2.5 (2.3)	3.4 (3.1)
Completeness (%)	92.7 (92.2)	100 (100)	91.9 (62.7)	94.0 (72.8)	95.9 (87.7)
R_merg_e (%)	5.3 (28.1)	11.2 (64.6)	5.7 (28.8)	5.9 (33.3)	14.4 (31.5)
Wilson B- value (Å^2^)	15.7	42.1	34.1	31.2	25.8
**Refinement**					
R_cryst_ (%)	14.9	24.6	20.6	19.6	25.1
R_free_ (%)	19.9	26.7	24	23.5	27.6
Number of protein atoms	8857	7773	14076	15196	14422
Number of CoA atoms	0	147	0	0	0
Number of substrate/inhibitor					
atoms	0	24	0	72	60
Other heteroatoms	4	2	0	2	2
Water molecules	875	147	715	1017	261
B-factor, Å^2^					
Overall	17.2	41.2	26	45.3	23.0
Protein	16.5	41	26.2	45.2	23.0
Ligands	-	67.5	-	52.9	27.3
Water	24.7	36.8	22.6	45.3	16.2
Rmsd from ideal geometry					
Bond length (Å)	0.024	0.012	0.008	0.008	0.011
Bond angles (deg.)	2.023	1.4	1.0	1.1	1.2
**Ramachandran Plot** (%)					
Residues in preferred regions	98	96.3	96.7	96.7	96.5
Residues in allowed regions	1.3	3.7	3.3	3.3	3.5
Outliers	0.7	0	0	0	0

Values in parenthesis are for the highest resolution shell.

A 1.6 Å diffraction dataset from a DapA crystal was collected at the beamline ID14:4 of the European Synchrotron Radiation Facility (ESRF, Grenoble, France) at 110 K. DapA crystals belong to the space group P1 with cell dimensions *a* = 43.1 Å, *b* = 51.7 Å, *c* = 140.3 Å, and *α* = 95.7°, *β* = 90.0°, *γ* = 113.3°. The statistics of the data set are given in Table 1.

### Molecular replacement and crystallographic refinement

The structure of *Pa*DapD was determined initially by molecular replacement in space group P4_1_2_1_2 using the program MOLREP [35]. A polyalanine model of the trimer of the putative tetrahydropyridine-2-carboxylate N-succinyltransferase from *Campylobacter jejuni* (PDB code 2RIJ) with the cofactor and the solvent atoms omitted was used as the search model. The best solution had a score of 0.295 and an R-factor of 55.9%. In order to monitor the refinement process, 5% of the X-ray data were removed for the calculation of R-free. Initial cycles of restrained refinement using Refmac5 [36] resulted in a drop of the R-factor by 15%. The correctness of the molecular replacement solution was confirmed by the appearance of electron density for the side chains specific for *Pa*DapD. Tight NCS restraints were applied throughout refinement for the three subunits in the asymmetric unit.

The structure of *Pa*DapD in the monoclinic crystal form was determined by MOLREP [35] using the refined coordinates for the *Pa*DapD trimer from the tetragonal crystal form. The P2_1_ crystals contain two trimers in the asymmetric unit. Since these crystals exhibited various fractions of twinning, twin refinement as implemented in Refmac5 [36] was employed. NCS restraints were used throughout the refinement. Isotropic B-factor refinement was used in all cases, except for the *Pa*DapD complex with L-2-aminopimelic acid. In this case TLS refinement was carried out in the final cycles, resulting in a decrease of R_free_ by 1.5%.

The structure of *Pa*DapA was determined by molecular replacement using the program MOLREP [35], and the coordinates from *Escherichia coli* DapA (2OJP) [37] as search model. The best solution contained four polypeptide chains in the P1 unit cell, with a MOLREP score of 0.401 and R-factor of 0.489. The program Arp/warp [38] was used in the initial model building and refinement steps.

Manual rebuilding of all models was carried out with the program Coot, based on sigma-A weighted *2Fo-Fc* and *Fo-Fc* electron density maps [39]. Examination and adjustment of the models were interspersed with rounds of refinement using Refmac5 [36]. In the structures derived from the monoclinic crystal form, i.e apo-enzyme, and the binary complexes with L-2 and D-2-aminopimelic acid, respectively, the C-terminal part of the protein is disordered, and residues 330–344 were not modeled. Water molecules were added based on peak heights, shape of the electron density, temperature factor and capability to form hydrogen bonds with surrounding protein residues and/or other water molecules. Well defined difference electron density allowed modeling of CoA and succinate for the CoA-succinate complex of *Pa*DapD. The final model of this complex contains three chains of DapD, amino acid residues 1–245 and 251–343, three CoA and succinate molecules and 147 water molecules. No electron density was observed for the loop comprising residues 246–250. The electron densities for the inhibitor/substrate molecules in the binary complexes are well defined in all six subunits of the asymmetric units, with the exception of subunit B of the DapD-L-2-AP complex. The difference electron density for a ligand in the active site of this subunit is not well defined, indicating low occupancy, and we therefore did not model any bound substrate.

At the final stage anisotropic B-factors were used in the refinement of the DapA structure as implemented in Refmac5. The final model of *Pa*DapA contains four polypeptide chains (chain A: residues 1–291, and chains B, C and D: residues 2–291), four chloride ions and 875 water molecules. Details of the refinement and protein models are given in Table 1. The crystallographic data have been deposited with the Protein Data Bank, accession codes 3R5A, 3R5B, 3R5C, 3R5D for *Pa*DapD and 3QZE for the *Pa*DapA.

Protein structures were analyzed and validated through the MOLPROBITY server [40]. Structural comparisons were carried out using the DALI algorithm [41] molecular contacts and interacting surfaces were analyzed with the PISA server [42]. Figures were made using the program Pymol (http://www.pymol.org).

## Results

### Characterization of *Pseudomonas aeruginosa* DapD

**Figure 2:**
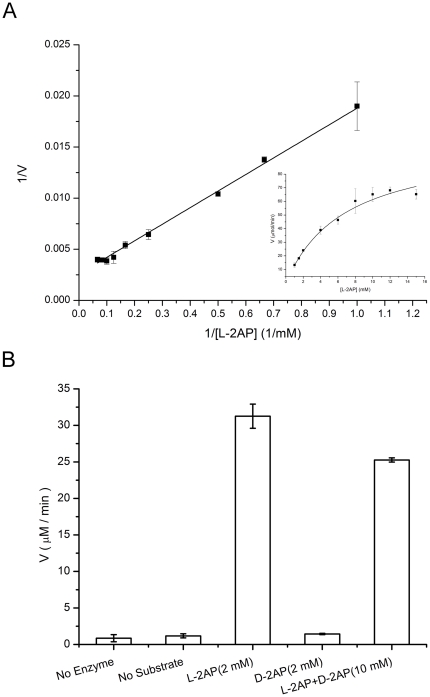
Substrate specificity and reaction kinetics for DapD from *P. aeruginosa*. A. Lineweaver – Burk plot for the dependence of DapD on the substrate L-2-aminopimelate. B. Activities of DapD in the presence of L-2-aminopimelate (L-2AP), D-2-aminopimelate (D-2AP) and a racemic mixture of the two compounds (L-2AP+D-2AP) as substrates.

DapD from *P. aerugionosa* PAO1 was expressed in *Escherichia coli* BL21(DE3) and purified to homogeneity. The size exclusion chromatography suggests a trimeric structure in solution, corresponding to a molecular mass of approximately 110 kDa. Recombinant *Pa*DapD was catalytically active as a succinyl transferase with L-2-aminopimelic acid and succinyl-CoA as substrates. Acetyl-CoA was not accepted as acyl-donor and magnesium ions were not essential for catalysis. Various amino-acids similar to 2-aminopimelate were tested, including L-lysine, adipic acid, α-amino-adipic acid, L-ε-acetyl-lysine, L-glutamate, L-glutamine, L-norleucine, but catalytic activity was only observed with 2-aminopimelate. The reaction is stereospecific with respect to the amino donor substrate, i.e. only L-2 aminopimelate was accepted as substrate with a K_M_ of 7.0 mM and a V_max_ of 105 µmol/min (figure 2A). D-2-aminopimelic acid is not accepted as a substrate by *Pa*DapD (figure 2B), but acts as a very weak inhibitor (IC_50_>20 mM).

### The structure of *P. aeruginosa* DapD

The structure of *Pa*DapD was determined by x-ray crystallography in its apo-form to 2.95 Å and in complex with coenzyme A and succinate to 2.3 Å resolution in the spacegroup P4_1_2_1_2. Incubation of the protein in a solution of 20 mM formyl-CoA for 15 minutes prior to crystallization resulted in a different crystal form belonging to the space group P2_1_ that reproducibly diffracted better. The structure of apo-*Pa*DapD was subsequently determined and refined to 1.8 Å resolution using data from this crystal form. In the tetragonal crystals (P4_1_2_1_2) the asymmetric unit contained a trimer of *Pa*DapD, while in the monoclinic crystal form (P2_1_) two such trimers were found in the asymmetric unit. In all structures of *Pa*DapD the oligomerization mode and fold were the same and resemble that of other DapD enzymes. Although the crystal packing interactions are different in the tetragonal and monoclinic crystals, the overall structure of the apo-*Pa*DapD trimer is essentially identical. Superposition of the individual subunits from different crystal forms typically gives rmsd values of 0.5 Å, and superposition of the trimers results in an rmsd of 0.55 Å.

**Figure 3:**
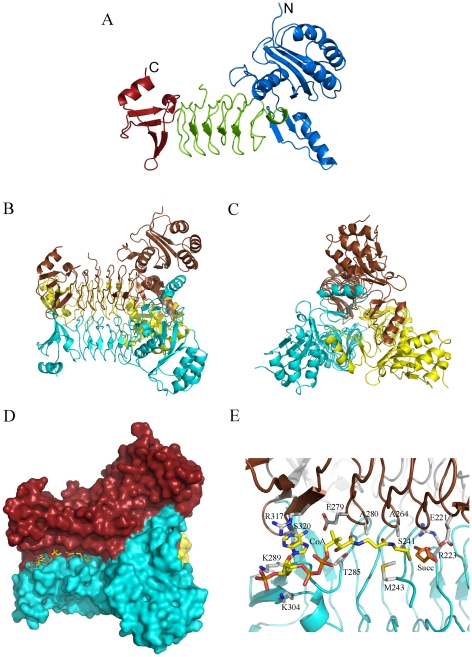
The structure of *P. aeruginosa* DapD. A. Cartoon of the subunit of tetrahydrodipicolinate N-succinyltransferase (DapD). The N-terminal domain is shown in blue, the central domain in green and the C-terminal domain in red. B & C: Views of the trimer of DapD. The three subunits are coloured blue, brown and yellow. D. Surface illustration of the trimer of the DapD-coenzyme A-succinate complex. The substrate binding grooves are formed between the left handed β-helix domains from adjacent subunits (blue and brown, respectively) of the trimer. Bound co-enzyme A and succinate are shown as stick models in yellow. E. Interactions of DapD with the bound coenzyme-A and succinate. The two subunits contributing to this active site are shown in blue and brown, respectively. Coenzyme-A is shown in yellow sticks and succinate in orange, while the amino acid side chains involved in the interaction are depicted in light gray.

**Figure 4:**
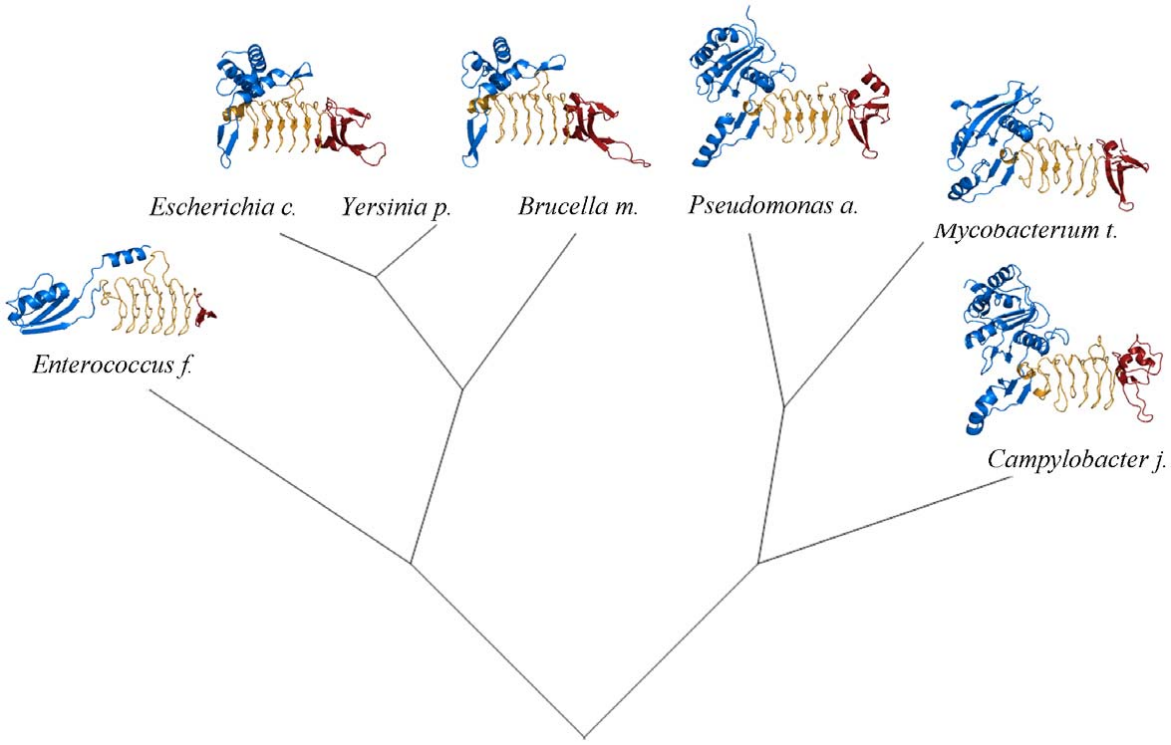
Evolutionary tree of DapD enzymes. The tree is based on a sequence alignment generated by the program Phylogenetic Tree available at http:www.cbrg.ethz.ch/services. Known crystal structures are added as illustrations to protein fold-evolution. The N-terminal domains are shown in blue, the central LβH-domains in orange and the C-terminal domains in red.

The subunit of *Pa*DapD consists of three domains, the N-terminal globular domain, a central domain, and a C-terminal domain (figure 3). The left handed β-helix (LβH) is the common feature in this protein family and builds up the central domain (residues 172–286) of each subunit. This domain also provides the majority of intrasubunit interactions of the trimer. The N-terminal domain consists of residues 1–171 and is rather large when compared to other members of the family (figure 4). It can be divided into two subdomains. The distal subdomain (residues 1–133) comprises a four-stranded anti-parallel β-sheet, flanked by three helices on one side and one helix on the opposite side of the sheet that faces the central domain. The medial subdomain (residues 134–171) comprises an α-helix and an anti-parallel β-hairpin and is involved in inter-subunit interactions as in other DapD structures known to date. The small C-terminal domain (residues 287–344) folds in a curved, four stranded anti-parallel β-sheet and two helices. This sheet extends the β-sheet of the central domain and contributes to trimer formation.

The trimer interface is rather large (figure 3) and about 1600 Å^2^ from each subunit are buried upon formation of the trimer. Approximately 50 residues from each subunit contribute to oligomerization. The trimer interface is predominantly polar with 26–28 hydrogen bonds and 12 salt bridges in every subunit contact area.

The left-handed β-helix fold occurs in a large number of proteins from viruses to plants and mammals. A Dali search using the atomic coordinates of *Pa*DapD identified several proteins with a similar β-helical domain, with the closest relatives being the putative DapD from *Campylobacter* (2RIJ) with a rmsd of 1.6 Å based on 307 Cα atoms and DapD from *M. tuberculosis* (3FSX) with 277 equivalent residues and an rmsd of 1.3 Å.

### The *Pa*DapD-CoA-Succinate complex

The active site of *Pa*DapD is located between neighbouring subunits (figure 3D), and two subunits are involved in the architecture of each active site. In each subunit interface a 35 Å long narrow crevice is formed where the reactants are bound. The residues contributing to the active site are from the central domain and the C-terminal domain, whereas the N-terminal domain is not directly involved in the formation of the active site.

Coenzyme A is bound in this narrow cleft formed at the interface of two adjacent subunits (figure 3D & E). Both subunits contribute to the interactions with CoA to a similar extent, with about 350 Å^2^ of the surface area of CoA buried by each subunit. CoA binds parallel to the trimer axis with the adenosine moiety positioned between the C-terminal domains of adjacent subunits. The pantetheine arm lies in the groove along the β-helix of two central domains and the β-mercaptoethylamine moiety is facing a cavity where the succinate molecule is bound (figure 3D & E). The position and binding mode of CoA is similar to the one found in *Mt*DapD and other acyl-CoA transferases, described as a “hooked” conformation of the adenosine moiety and an extended conformation of pantetheine arm [43]. The distance between the ribose-3′ phosphate and sulphur atom is 21.5 Å.

The adenine base of Coenzyme-A is inserted between the guanidium group of Arg317(A) of one chain and Gly287(C) of a neighbouring subunit. The carbonyl of Gly287(C) is at hydrogen bond distance with the ribose 2′ hydroxyl group. The 6′ amino group of the base is hydrogen-bonded with the main chain carbonyl oxygen atom of Arg318(C) and the side chain oxygen atom of Glu279(C). The backbone amino group of Ser320 interacts with the N1 nitrogen of the adenine ring. The 3′ phosphate group forms hydrogen bonds to the ε-amino moieties of Lys289(A) and Lys304(A). The pyrophosphate is accessible to the solvent (figure 3E) and does not form any interactions with the protein. The pantheteine oxygen atoms form hydrogen bonds to the main chain nitrogen atoms of Asn264 and Asn280.

Binding of CoA to *Pa*DapD does not induce any large conformational changes such as large scale domain re-arrangements as indicated by the r.m.s.d. value upon superposition of the subunits from the apo- and CoA complex of 0.55 Å. However CoA binding causes a disorder-order transition in the enzyme. In all structures of *Pa*DapD that lack bound CoA, the last 15 residues including an α-helix, are not resolved in the electron density, whereas they are well defined in the *Pa*DapD-CoA complex.

The crystal structure of the complex also contains a succinate molecule bound next to the acceptor arm of the CoA in the active site cleft (figure 3E), most likely due to the presence of 0.4 M succinate in the crystallization buffer. Bound succinate interacts with residues from both chains that make up the active site. One of the carboxyl groups forms hydrogen bonds with the guanidium moiety of Arg223 and the side chain of Ser241. The second carboxyl group interacts with the side chain of Glu221 and the main chain carbonyl of Gly238. This pattern of hydrogen bonds suggests however that one of the carboxyl groups of succinate or the carboxyl group of Glu221 is protonated. The position of the bound succinate corresponds well to the site of the succinyl-moiety in the ternary complexes of *Mt*DapD and *Ec*DapD with the co-substrate succinyl-CoA or its analogue, succinamide-CoA [26], [29]. With the exception of the hydrogen bond to the side chain of Glu221, the interactions between the enzyme and the bound succinate in *Pa*DapD are identical to those described for the succinyl moiety in the *MtDapD and EcDapD* complexes.

### The *Pa*DapD -2-aminopimelate complex structures

Several attempts were made to obtain crystals of *Pa*DapD with the substrate L-2-aminopimelate or its isomer D-2-aminopimelate without success, possibly due to the high concentrations (0.4–0.6 M) of succinate in the crystallization buffer. Eventually, these complexes could be prepared by soaking of monoclinic crystals of *Pa*DapD in crystallisation liquor, where succinate had been replaced with either L- or D-2-aminopimelate.

**Figure 5:**
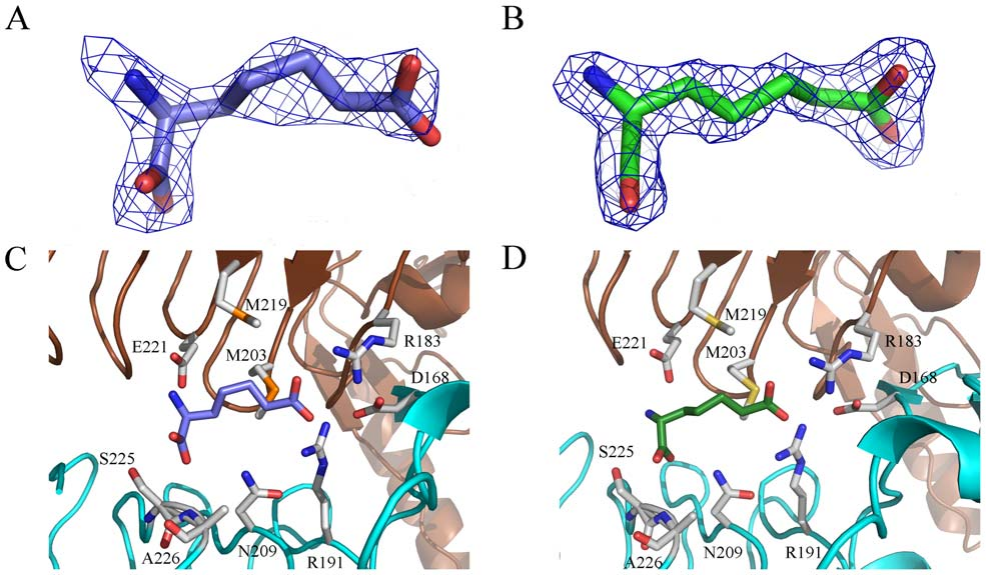
Ligand binding to *Pa*DapD. A & B: OMIT electron density maps [44] for bound ligands L-2AP (A) and D-2AP (B), contoured at 1.6σ. C & D: Interactions of L-2AP (C) and D-2AP (D) at the active site of *Pa*DapD. The two adjacent subunits that form an active site cleft are shown in blue and brown colours. L-2AP is depicted in blue and D-2AP in green. Enzyme residues interacting with the ligands are shown in light grey.

The binding site of L- and D-2-aminopimelate in *Pa*DapD is located in the narrow groove formed between two subunits, and is adjacent, but distinct from the binding sites for CoA and succinate (figure 5). D-2-aminopimelate is bound to *Pa*DapD in an extended conformation, well defined in the electron density maps (figure 5). In five of the six active sites in the crystal asymmetric unit the interactions of this ligand are identical. The ε-carboxyl group of D-2-AP forms hydrogen bonds to the guanidinium groups of the two arginine residues Arg181 and Arg193, and the appropriate orientation of the side chains of these residues is ensured by a salt bridge to the side chain of Asp168. The head α-carboxyl group is anchored to the protein by hydrogen bonds to the main chain amino group of Ala226 and the side chain of Asn209. The α-amino group of the ligand is within hydrogen bonding distance to one of the carboxylate oxygen atoms of Glu221. In the active site located at the interface between subunits A and B, the binding mode of the ligand is different. While the tail carboxyl group and the carbon chain of D-2-AP interact in a similar manner with the enzyme as in the other subunits, the binding modes of the α-carboxyl- and α-amino groups are altered. The α-carboxyl group occupies the position of the amino group observed in the majority of the active sites and is within hydrogen bonding distance to the carboxyl group of Glu221, indicating that one of these moieties is protonated. The amino group points towards the side chain of Asn209 and the main chain amino nitrogen atom of Ala226, but does not form any hydrogen bonding interactions. There are no indications in the electron density maps during refinement that the active sites contain mixtures of these two binding modes, and the reason why one of the active sites specifically selects this ligand conformer remains unclear.

The crystallographic binding studies with the amino donor substrate L-2-AP allowed straightforward fitting of the substrate in the active sites of all subunits with the exception of chain B, where the electron density is too weak for unambiguous modelling of bound L-2-AP, indicating lower occupancy of the ligand. In three of the subunits (chains A, D and E) the binding mode of the substrate is very similar to those seen with its stereoisomer, D-2-aminopimelate. The ε-carboxyl group of L-2AP forms salt bridges with the guanidinium groups of residues Arg18 and Arg19 and the α-carboxylate of L-2AP forms hydrogen bonds to the side chain of Asn209 and the main chain amino group of Ala226. In addition, the α-amino group of L-2AP is also within hydrogen binding distance to the side chain of Glu221. A comparison of the *Pa*DapD-L-2AP binary complex with the structure of the ternary complex of the *E. coli* enzyme with coenzyme A and L-2AP [29] reveals a very similar binding mode and conserved enzyme-ligand interactions in both enzymes.

The binding of the substrate to the remaining two active sites (C and F) is somewhat different from the one observed in chains A, D, and E and can best be described as intermediate binding steps. While the tail carboxyl group and parts of the alkyl chain of L-2-AP are inserted into the active site, with the interactions of the carboxyl tail to the two arginine residues Arg181 and Arg193 formed, the remaining part of the carbon chain and the head group is still in contact with the bulk solution and does not yet interact with protein residues. Thus, the ensemble of enzyme-ligand structures of *Pa*DapD seen in the asymmetric unit might represent snapshots of the binding process with binding to chain F and C just after insertion of the ε-carboxyl group and chains A, D and E as the final steps with most or all interactions in place.

### Intratracheal lung infection of NMRI mice with *P. aeruginosa* and the DapA knock-out strain

For validation of DapA as potential target for novel drugs against *P. aeruginosa* infections we attempted to create single gene knockout strains of *P. aeruginosa* PAO1.

**Figure 6:**
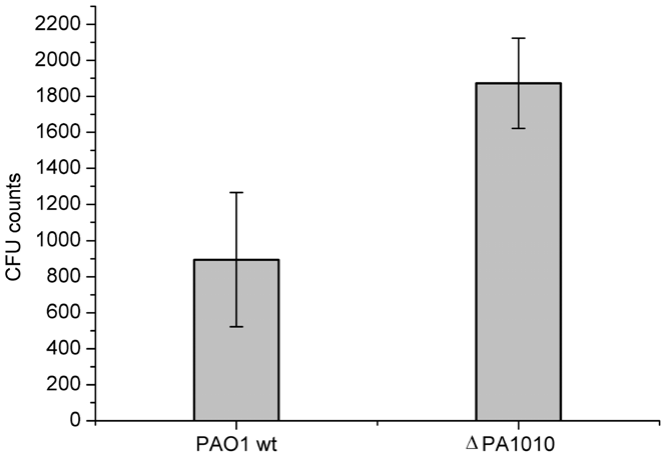
Assessment of the effect of PA1010 gene deletion *in vivo*. Quantification of *P. aeruginosa* colonies grown in the lung of mice intratracheally infected with agarose beads loaded with the *P. aeruginosa* gene knock-out mutant (ΔPA1010) in comparison to PAO1 wildtype (wt).

After three conjugations and screening of about 100 transconjugants one *dapA* KO mutant was obtained and confirmed by sequencing. NMRI mice were intratracheally infected with agarose beads loaded with *P. aeruginosa* PAO1Δ*dapA* as described in the method section. Mice were separated in groups per ten animals each. The single groups were infected with bacterial beads loaded with the mutant *P. aeruginosa* PAO1Δ*dapA* and PAO1 wild type as control. 72 hours post infection lung homogenates were plated in different dilutions for quantification of the bacterial load. The mutant *P. aeruginosa* PAO1Δ*dapA* did not show decreased numbers of total bacteria counts 72 hours after infection compared to the wild type strain (figure 6).

### Structure of *Pseudomonas aeruginosa* DapA (PA1010)

**Figure 7:**
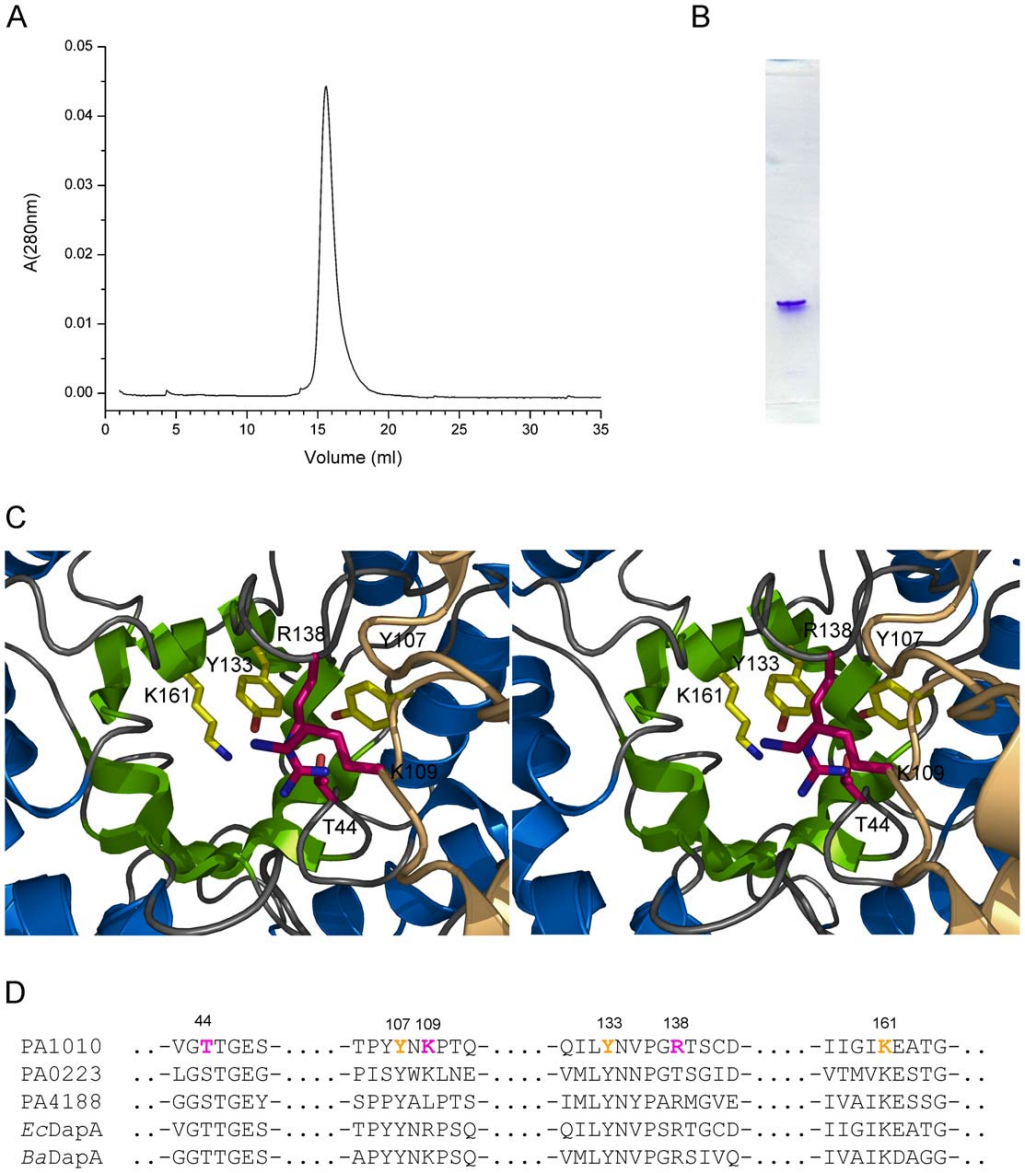
The structure of *P. aeruginosa* DapA. A. Size exclusion chromatography elution profile of *Pa*DapA (PA1010) indicating that a single species exists in solution. Based on the calibration curve (insert) the calculated molecular mass is 60 kDa. B. *Pa*DapA (PA1010) purified sample analyzed in native polyacrylamide gel electrophoresis indicating a single species. C. Stereo view of the active site of *Pa*DapA located in the center of the α/β barrel. Amino acid side chains forming the active site are indicated as stick models. Residues conserved in the three homologues PA1010, PA0223 and PA4188 are shown in yellow, while the variable positions Thr44, Arg138 and Lys109 are indicated in purple. D. Sequence conservation in DapA enzymes from *Escherichia coli*, *Bacillus anthracis*, *Pseudomonas aeruginosa* and the proposed DapA paralogues in the PAO1 genome PA0223 and PA4188. The active site residues in *Pa*DapA (PA1010) are indicated with yellow or purple colour as in C.

The crystal structure of *Pa*DapA was determined by X-ray crystallography to 1.6 Å resolution. In contrast to most DapA enzymes from Gram-negative bacteria *Pa*DapA is not a homo-tetramer but exists as a homodimer in solution and in the crystal. Size exclusion chromatography suggested only one species with a MW of 60 kDa, i.e. a dimeric structure. This finding is also consistent with native polyacrylamid gel electrophoresis, which only showed one species present in solution (figure 7). *Pa*DapA is also a dimer in the crystalline state, where the buried surface areas at the dimer interface are 1500 Å^2^, whereas contacts to other subunits in the crystal lattice involve surfaces of 300–400 Å^2^. The observation of a dimeric subunit arrangement in the crystals is consistent with the results obtained by size exclusion chromatography in solution (data not shown). The basic fold of the subunit is an (β_/_α)_8_ barrel with a C-terminal extension of approximately 60 amino acid residues folded into an additional small α-helical domain. Comparison to the structure of *Pa*DapA (PDB: 3PS7) determined recently, albeit at the significantly lower resolution of 2.9 Å [20], gives an rmsd value of 0.3 Å for 290 aligned Cα atoms.

### Characterisation of *P. aeruginosa* DapA (PA1010) and its homologue, PA0223

The enzymatic assays using the recombinant gene product of PA1010 verify that this enzyme functions as DapA, as the coupled assay with DHDPR and S-ASA and pyruvate dependent dihydrodipicolinate-synthase results in a specific activity of 21 µmol/min mg(protein) at 22°C. This value is comparable to the activity of DapA from *E. coli* [23].

The two DapA homologues with unknown function found in the *P.aeruginosa* PAO1 genome, PA0223 and PA4188 show high levels of conservation in the active site. The heterologous expression of PA4188 gave insoluble protein product and could not be tested. PA0223 was purified utilizing the N-terminal His6-tag and its folding integrity was confirmed by circular dichroism spectroscopy. PA0223 did however not show any activity as dihydrodipicolinate-synthase, even when tested at significantly higher concentrations than PA1010 (DapA) in the assays.

## Discussion

The structure of *Pa*DapD shows the typical features of this fold family, a left-handed parallel β-helix (LβH) domain, with additional N- and C-terminal extensions (figure 4). The N-terminal domains show large variations in structure among the DapD enzymes although the substrates and the reaction catalyzed are identical [26]. Likewise, the C-terminal domains show variations in length, from about 20 to 70 residues, and consequently range from a small β-hairpin addition to the LβH-module in *Enterococcus facalis* to a complete domain with its own hydrophobic core as in DapD from *M. tuberculosis* [26] and *E. coli* [25]. *Pa*DapD shows distinct features at the N- and C terminal domains that are structurally different from those described for DapD enzymes from Gram-negative bacteria and shows more similarity to DapDs from *M. tuberculosis* and *C. jejeuni* (figure 4).

### Structural basis of stereoselectivity

*Pa*DapD is specific for L-2AP and the D-isomer is a weak inhibitor of the enzyme, with an IC_50_>20 mM. The crystal structures of *Pa*DapD with L-2AP and D-2AP show that both compounds are recognized by the enzyme, bind at the same binding site and interact with the same enzyme residues. The high K_i_ value of D-2AP does not allow a more detailed kinetic characterisation of the mode of inhibition, but the fact that substrate and inhibitor bind at the same site would suggest a competitive inhibition mode.

**Figure 8:**
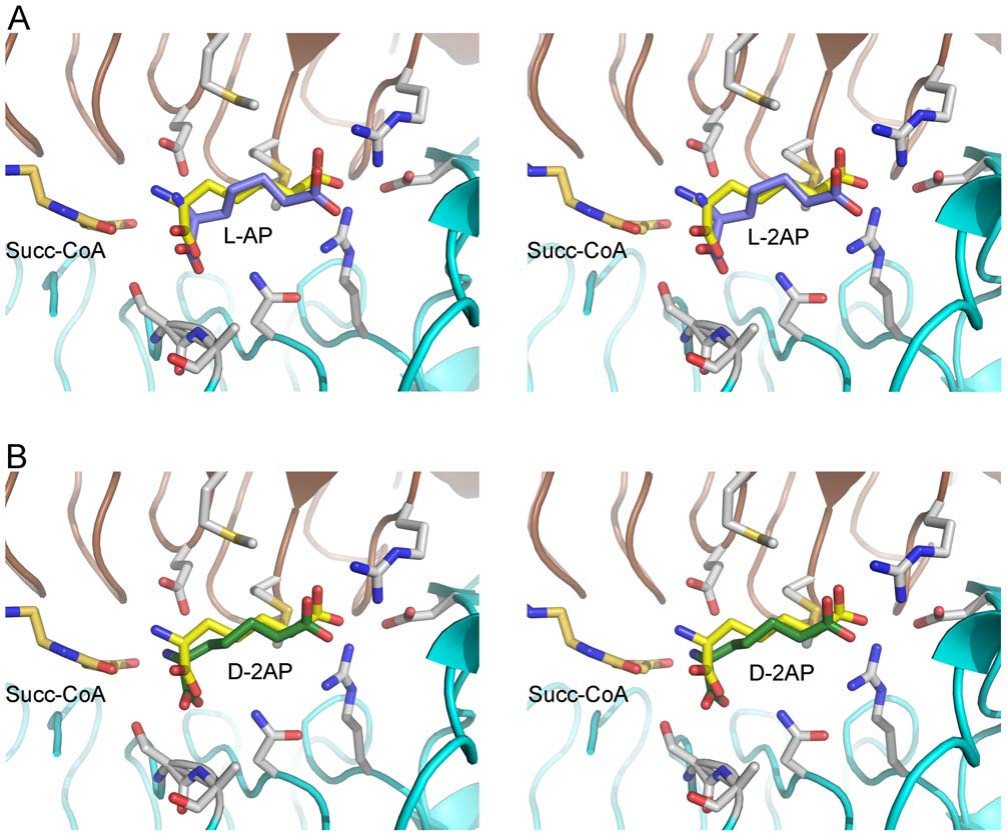
Structural basis of inhibition by D-2-aminopimelate. A. Composite stereo view of the active site of *Pa*DapD with bound succinamide-CoA, and the substrate L-2-AP. The position of the inactive substrate analogue succinamide-CoA (orange) and the L-2-aminopimelate (yellow) bound in the ternary complex were derived from a superposition with the DapD-succinamide-CoA-L-2AP complex (1KGQ). The bound L-2AP in the *Pa*DapD-L-2AP complex is shown in blue. B. View of a composite model of the catalytically incompetent complex of *Pa*DapD with bound succinamide-CoA, and the inhibitor D-2AP. The model was created using the same templates as in (A), the bound D-2AP in the structure of the complex of *Pa*DaD with this ligand is shown in green.

As both isomers of 2-AP bind to the enzyme active site the question arises as to why only the L-form serves as substrate. In the absence of a crystal structure of a catalytically competent ternary complex with enzyme, succinyl-CoA and substrate, we have modelled a Michaelis complex (figure 8) based on superposition of the structures of the *Pa*DapD-CoA-succinate and *Pa*DapD – L (or D) -2AP complexes with that of the structure of DapD-succinyl-CoA-pimelate and DapD-succinamide-L-2-AP [29]. In such a model, the amino group of L-2-AP points towards the carbonyl group of succinyl-CoA and is poised for a nucleophilc attack on this carbon atom, i.e. for succinate transfer (figure 8A). The interaction with the side chain of Glu221 could contribute to rate enhancement by deprotonation of the substrate amino group as was proposed for the *E. coli* enzyme [28], [29]. A corresponding model of the inhibited ternary complex shows that the amino group of D-2AP is pointing in a direction not favourable for nucleophilic attack on succinyl-CoA (figure 8B). We therefore suggest that binding of D-2-AP in a conformation incompatible with catalysis is the underlying basis for the discrimination of the enzyme against this stereoisomer of 2-aminopimelate.

The *dapA* gene is not essential for infection in mice, as shown by the ability of the *P. aeruginosa* Δ*dapA* mutant to grow in a mouse acute infection model. A post-mortem analysis using structural information for *Pa*DapA and the genome of *P. aeruginosa* identified two other orfs that may represent DapA homologues. PA0223 (Uniprot id: Q9I6R5) is a tetrameric enzyme (PDB:3NA8, unpublished) that shows 30% sequence identity and 1.5 Å rmsd over 291 aligned Cα atoms when comparing to *Pa*DapA (PA1010). PA4188 is an uncharacterized protein (Uniprot id: Q9HWJ3) that shows 27% sequence identity to *Pa*DapA (figure 7). Enzymatic assays using recombinant PA0223 show that this protein does not act as a dihydropicolinate synthase *in vitro*, making it unlikely that it can replace DapA (PA1010) in the PAO1Δ*dapA* mutant. The reason for the ability of the PA1010 knockout mutant to grow in the lungs of infected animals remains unclear, but our data suggest that DapA is not a promising target for the design of novel antibacterials against *P. aeruginosa*.
